# Inflammaging: Implications in Sarcopenia

**DOI:** 10.3390/ijms232315039

**Published:** 2022-11-30

**Authors:** Eduardo Antuña, Cristina Cachán-Vega, Juan Carlos Bermejo-Millo, Yaiza Potes, Beatriz Caballero, Ignacio Vega-Naredo, Ana Coto-Montes, Claudia Garcia-Gonzalez

**Affiliations:** 1 Instituto de Investigación Sanitaria Del Principado de Asturias (ISPA), Av. Del Hospital Universitario, 33011 Oviedo, Spain; 2 Department of Morphology and Cell Biology, University of Oviedo, 33006 Oviedo, Spain; 3 Instituto de Neurociencias del Principado de Asturias (INEUROPA), 33006 Oviedo, Spain

**Keywords:** inflammation, aging, sarcopenia, skeletal muscle

## Abstract

In a world in which life expectancy is increasing, understanding and promoting healthy aging becomes a contemporary demand. In the elderly, a sterile, chronic and low-grade systemic inflammation known as “inflammaging” is linked with many age-associated diseases. Considering sarcopenia as a loss of strength and mass of skeletal muscle related to aging, correlations between these two terms have been proposed. Better knowledge of the immune system players in skeletal muscle would help to elucidate their implications in sarcopenia. Characterizing the activators of damage sensors and the downstream effectors explains the inference with skeletal muscle performance. Sarcopenia has also been linked to chronic diseases such as diabetes, metabolic syndrome and obesity. Implications of inflammatory signals from these diseases negatively affect skeletal muscle. Autophagic mechanisms are closely related with the inflammasome, as autophagy eliminates stress signaling sent by damage organelles, but also acts with an immunomodulatory function affecting immune cells and cytokine release. The use of melatonin, an antioxidant, ROS scavenger and immune and autophagy modulator, or senotherapeutic compounds targeting senescent cells could represent strategies to counteract inflammation. This review aims to present the many factors regulating skeletal muscle inflammaging and their major implications in order to understand the molecular mechanisms involved in sarcopenia.

## 1. Introduction

Increased lifespan during the last few centuries led to an increase in human longevity and, consequently, to age-related pathologies [1]. In the past, efforts to increase longevity resulted in life extension at the expenses of life quality [2]. More recently, the importance of increasing lifespan without losing quality of life has been emphasized, as featured in [1]. This points out the relevance of increasing endeavors delaying the physiological change that results in age-related disease and disability.

The World Health Organization (WHO) has defined healthy aging as a process of maintaining functional capacity to enable well-being in old age [3]. The elderly’s health status becomes a priority, emphasizing that action is urgent [4]. Finding compensatory responses to the hallmarks of aging by further understanding the physiological sources of aging-causing damage becomes a demanding need in the contemporary world [5].

Frailty can be considered as a clinically recognizable state of increased vulnerability, as a consequence of a reduced functional reserve, which results in increased vulnerability to stressors and a limited capacity to maintain homeostasis [6]. It is characterized by unintentional weight loss (10 lbs/4.5 kg in past year), self-reported exhaustion, weakness (grip strength), slow walking speed, and low physical activity, and serves as an indicator of functional impairment [7]. Malfunction of skeletal muscle results in compromised functional performance and increased frailty.

Skeletal muscle is the most abundant tissue in humans, constituting 40–50% of their body mass. It is vital for basic functions such as eating (chewing and swallowing), breathing (inhaling and exhaling) and maintaining body posture (static and dynamic), making it essential for independent living. Sarcopenia is defined as a progressive and generalized skeletal muscle disorder implicating accelerated loss of muscle mass, strength and function [8,9]. It is associated with increased adverse outcomes, including balance problems, falls, functional decline, frailty and mortality [10]. Loss of mobility is one of the major consequences of age-related skeletal muscle deterioration and the primary cause of independency decline [11]. Additionally, sarcopenia is associated with worse hospitalization recovery of physical functioning, lowers the rate of discharge and increases the dependency level after hospitalization [12,13].

The elderly have a high risk of sarcopenia, which would induce changes in muscle mass, structure and function. Sarcopenia is a multifunctional disease, and the underlying pathophysiological mechanisms are still poorly understood [14]. Among the many molecular changes associated with old age, multiple factors—including immobility, malnutrition, low protein intake, changes in hormones and metabolism, systemic inflammation and neuromuscular aging—may underlie age-induced sarcopenia [15,16]. Skeletal muscle cells are high specialized cells with a complex organized structure, presenting a dense packaging of contractile proteins and organelles. This organized structure implies that protein and organelle turnover has a major impact on myofiber size and functionality, and that perturbations of the normal homeostatic turnover greatly affect skeletal muscle performance [17]. As a tissue with high energetic needs, a blunted anabolic response along with systemic conditions such as inactivity, infections, diabetes, organ failure or inflammatory diseases, all contribute to promoting muscle atrophic condition accompanied by fat infiltration and fibrosis [18,19].

Oxidative stress and inflammation are two hallmarks of age-related muscle atrophy and are potential targets for therapeutic approaches [20]. A growing number of studies are highlighting inflammation as a crucial regulator of the mechanisms controlling skeletal muscle homeostasis and ultimately leading to sarcopenia [21,22,23]. The term “inflammaging” refers to systemic, chronic, sterile, low-grade inflammation observed in many elderly individuals, which is associated with increased risk of various diseases [24,25]. It is thought to be the long-term result of chronic physiological stimulation of the innate immune system, mainly caused by the dysregulation of the cellular receptors of self and non-self [26,27].

Although enhanced immune responses are beneficial during early life and in adulthood, an uncontrolled activation during aging becomes detrimental. Chronic subclinical inflammation has been linked to the aging process, in which dysfunctional cellular repair mechanisms trigger the activation of proinflammatory pathways. Inflammaging becomes a risk factor for cardiovascular diseases, diabetes, chronic kidney disease, osteoporosis and neurological diseases [28,29,30,31,32]. Additionally, chronic inflammation has been related to sarcopenic hallmarks such as increased skeletal muscle wasting, strength loss and functional impairments [21,33].

In this review, we describe the role of low-grade systemic inflammation in skeletal muscle. In the following sections, we summarize the main molecular and cellular mechanisms underpinning aging-associated inflammation in sarcopenia. Finally, as strategies to prevent age-associated muscle changes, we suggested the role of senolytics and melatonin as possible interventions. 

## 2. Aging Factors in Skeletal Muscle (*When You Are Old and Gray*)

Striated skeletal muscle is highly organized tissue constituted of several bundles of muscle fibers known as myofibers. Each myofiber is in turn formed by several myofibrils and, surrounding the fibers is a plasma membrane called the sarcolemma. Muscle fibers are embedded into a net of connective tissue formed by an extracellular matrix (ECM) consisting of a mesh of collagenous components and other macromolecules. The skeletal muscle connective tissue also contains different cell populations, including fibroblasts and immune cells, as well as nerves and blood vessels. A complex network encompassing all these components allows the synchronized contraction of the organ.

Any change in the functional capacities and synergistic functionality of the many cell types populating skeletal muscle may compromise its performance and repair. Environmental factors such as inflammation and oxidative stress contribute to muscle pathophysiological responses such as hypertrophy, atrophy and fibrosis [34]. Hypertrophy is characterized by the growth of muscle mass due to an increase in fiber size, with or without an increment of fiber numbers (hyperplasia). Adaptive hypertrophy occurs in response to exercise, as the myofibers accumulate more structural contractile proteins. However, under pathological circumstances such as muscle injury or damage, hypertrophy acts as a compensatory mechanism. In this case, pathological hypertrophy is followed by atrophy, as in the case of Duchenne muscular dystrophy [35]. Muscle atrophy occurs when protein degradation rates exceed protein synthesis. Two major protein degradation pathways are involved in this process: the proteasomal and the autophagic pathways [36]. Atrophy ultimately leads to muscle wasting. Among the other proteins involved in these degradation pathways, Atrogin-1 and MURF1, two ubiquitin E3 ligases of the proteasome system, are widely recognized as master regulators of muscle atrophy [37]. On the other hand, fibrosis is a consequence of dysregulation of the turnover between ECM synthesis and degradation, and it is characterized by uncontrolled and irreversible expansion of the ECM components at the expense of myofibers. Skeletal muscle fibrosis impairs muscle function and disrupts muscle regeneration [38]. Oxidative stress and inflammation act in a feedback loop, contributing to these pathologies [39].

Another characteristic of skeletal muscle is its high metabolic capacity, which is essential for the generation of ATP required for the contraction of the myofibrils that generates movement and force. Two processes are involved in generating energy: glycolysis and oxidative metabolism. As a consequence of these high energetic requirements, the alteration of metabolic processes affects muscle functionality. Muscle metabolism involves both muscle fibers and immune cells, indicating a crosstalk between these cell types [40].

Age-related reduction in muscle repair efficiency contributes to the development of sarcopenia [10]. Indeed, age-related features such as cellular senescence, oxidative stress, mitochondrial dysfunction, reduced numbers and regenerating capacity of satellite cells, adipose tissue accumulation, inadequate nutrition, hormonal and cytokine changes all play a strong role in the development of sarcopenia. For its part and importantly, the immune system has pivotal functions in controlling all these age-related features. Thus, muscle wasting is a multifactorial condition in which a feedback process between different players, and inflammation is at the center of it. Identifying all the relevant age-dependent factors affecting muscle biology and their synergies will help to develop strategies to counteract or at least limit the inefficiency of muscle repair arising with aging.

## 3. Decline of Muscle Stem Cells (*Running out the Gold Mine of Skeletal Muscle Regeneration*)

Skeletal muscle regeneration is a highly orchestrated process requiring the simultaneous activation of various cellular types and molecular pathways. Mammalian regeneration of skeletal muscle is mediated by muscle stem cells (MuSCs), also called satellite cells, the primary stem cells of adult skeletal muscle. In adult muscle, upon homeostatic conditions, MuSCs are in a quiescent state and only upon activation of extrinsic signaling pathways do they activate and begin proliferating [41,42]. Muscle regeneration requires four sequential but overlapping stages. First, the reception of signaling from necrotic or damaged muscle fibers (or from other surrounding sources of damage). Second, the recruitment of immune cells to the local sites of damage. Third, the activation, differentiation, and fusion of MuSCs. Fourth, the maturation and remodeling of newly formed myofibers [42]. During regeneration, the myogenic lineage is regulated by a hierarchy of transcription factors corresponding to these four phases [34]. Alterations in any of these phases compromise the regenerative capacity [43].

Quiescent MuSCs present the capacity to re-enter the cell cycle and proliferate, while a small subpopulation return to quiescence to maintain the stem pool [44]. The ability of skeletal muscle to regenerate is tightly controlled by the interaction between MuSCs and their microenvironment, also known as MuSC niche [45]. Upon injury, a well-orchestrated time-dependent inflammatory response accompanies and regulates MuSC behavior [46]. During the acute inflammation phase, neutrophils attend to the injury site, followed by macrophages, whose main role is to engulf cellular debris and to secrete several cytokines that stimulate MuSCs. Two types of macrophages attend sequentially. First the M1, a subtype producing pro-inflammatory cytokines and stimulating MuSC activation and proliferation. Eventually, the expansion of M2 macrophages is associated with tissue repair and satellite cell differentiation via production of anti-inflammatory cytokines. The process requires final resolution of all types of immune cells.

Chronic inflammation associated with myopathies, obesity and aging creates a non-favorable niche for MuSC renewal [47]. Transition from M1 to M2 macrophage phenotype and their correspondent cytokine release during skeletal muscle regeneration is a key factor for proper muscle regeneration [48]. An imbalance of M1–M2 macrophages patterns was observed in aging and disused skeletal muscle [49]. In another study, an increase in M2 macrophages during aging is associated with increased intermuscular adipose tissue and impairment of muscle metabolism [50]. The interplay between macrophages and MuSCs was also studied in Duchenne Muscular Dystrophy (DMD), a primary myopathy caused by the loss of functional dystrophin protein, where sustained myogenic activation of MuSCs can be alleviated by M1-to-M2 induction via IL-10 administration by reducing the activation of the M1 phenotype [51]. Moreover, depletion of macrophages in DMD and in acutely injured muscles impairs muscle regeneration [52,53].

A decrease in expression of interferon-gamma (IFN -γ)-related genes in macrophages of aged mice impairs MuSC activation and promotes fibrosis in a reversible fashion. For instance, administration of Cytokine C-X-C motif chemokine 10 (CXCL10), a molecule secreted by IFN-responsive macrophages, promotes proliferation and differentiation of MuSCs via interaction with the CXCR3 receptor, leading to increased cross-sectional areas and decreased fibrosis [54].

The age-related defects in both MuSCs and their niches result in a fibro-adipogenic phenotype characteristic of aged and dystrophic muscle. Chronic activation leads to stem-cell exhaustion through an imbalance of stem-cell quiescence and proliferation [55]. On the other hand, in aged muscle, satellite cells experiencing prolonged quiescence begin to express senescence markers and present detrimental effects as elevated proteotoxic stress, mitochondrial dysfunction, genomic instability and impaired regeneration [56]. Geriatric MuSCs are not capable of transitioning from G0 quiescence to activation. Importantly, de-repression of p16^INK4a^ (Cdkn2α) in pre-senescent MuSCs acts by inhibiting quiescence-inducing pathways and increasing DNA damage [57]. These data indicate that healthy MuSCs in aging require a balance between activation and quiescence, and that niche conditions mainly driven by chronic inflammation secretory factors are regulating this process [58,59].

Elevated activity of the p38α and p38β mitogen-activated kinase pathway in MuSCs increases the expression of senescence markers upon aging, and these changes cannot be reverted by transplantation into a young microenvironment [60]. This indicates that muscle regeneration is impaired with aging, at least in part, by a cell-autonomous functional decline in MuSCs [60]. The increase in p38α activity is mediated by tumor necrosis factor (TNF) activation linking inflammation to maintenance of regenerative capacity [61].

Another pathway by which macrophages could contribute to muscle wasting associated with aging is the Prostaglandin E_2_ (PGE_2_) pathway. PGE_2_ expression is reduced in aged murine skeletal muscles and myofibers. At the time, tissue-resident macrophages are the source of 15-hydroxyprostaglandin dehydrogenase (15-PGDH). Inhibition of 15-PGDH prevents muscle atrophy and markedly increases the cross-sectional area of myofibers, muscle mass, strength and endurance by decreasing transforming growth factor–β (TGF-β) signaling, the ubiquitin proteasome pathway and increasing mitochondrial biogenesis [62].

Taken together, the data suggest that age-dependent dysregulation of the immune system and, in particular, of macrophages, affects MuSCs’ potential to efficiently renew and regenerate skeletal muscle.

## 4. Impairment of Damage Sensors and Increase in Alarm Signaling (*When the Enemy Is Within*)

The innate and adaptive immune systems protect our cells from potentially harmful agents. These agents can be categorized into “non-self” (bacteria, viruses, fungi and parasites),“self” (endogenous cell debris and misplaced or misfolded molecules) or “quasi-self“ (nutrients and gut microbiota) [63]. These stimuli interact with pattern recognition receptors (PRRs), including Toll-like receptors (TLRs), nucleotide-binding oligomerization domain-like receptors (NLRs), C-type lectin receptors (CLRs) and RIG-1 like receptors (RLRs). PRRs can recognize conserved molecular structures produced by non-self molecules, including viral and bacterial products (PAMPs—pathogen-associated molecular patterns), endogenous molecules (DAMPs—damage-associated molecular patterns) and nutritional and metabolic products from the gut microbiota (classified as quasi-self) [63]. As a consequence of the recognition by PRRs, activation of signaling cascades takes place, involving the nuclear factor-κB (NF-κB), activator protein-1 (AP-1) and mitogen-activated protein kinase (MAPK) pathways, among others. These pathways activate the production of pro-inflammatory cytokines/chemokines such as interleukin-1 (IL-1), tumor necrosis factor-α (TNF-α) and interleukin-6 (IL-6), triggering inflammation [64].

Muscular dystrophies are characterized by enhanced tissue damage and consequent necrosis, which releases signal molecules that can be sensed by PRRs triggering exacerbated and sustained inflammatory responses [65,66,67]. Exaggerated activation of the cell-surface receptors TLR2/4 and TLR7/8/9, which are associated with intracellular endosomes, is mainly responsible for the chronic activation of the innate immune system in skeletal muscle, which leads to a perpetuated pro-inflammatory state and damage [66,67].

Recently, mitochondrial DNA (mtDNA) has been recognized as a novel source of DAMPs that can potentially elicit inflammation, even while the subsequent consequences can differ. It has been shown that muscle crush injury releases mitochondrial DAMPs, causing inflammatory responses through innate immune pathways identical to those activated in sepsis, involving formyl peptide receptor-1 and TLR9 [68]. In particular, optic atrophy 1 (Opa1), an inner mitochondrial membrane protein involved in regulating mitochondrial stability, plays an essential role in activating this pathway. Opa1 deficiency in muscle leads to the engagement of TLR9, resulting in the activation of the NF-κB inflammatory program, which contributes to enhanced Fgf21 expression and growth impairment [69]. Additionally, cytosolic mtDNA engages the DNA sensor cGAS and promotes STING-IRF3-dependent signaling to promote interferon-stimulated genes (ISG) expression [70]. Consistent with these observations, human patients with sarcopenia display increased levels of circulating cell-free mtDNA, positively correlating with plasma interleukin IL-6 and IL-8 [71].

Patients in the critically ill intensive care unit (ICU) often develop muscle atrophy and weakness with increased morbidity and mortality. Serum amyloid A1 (SAA1), a protein associated with inflammation and acute-phase responses during sepsis, which is secreted by the liver, but also by skeletal muscle, causes myocyte atrophy by activating the TLR2/TLR4/NF-κB/p65 signaling pathway [72]. Furthermore, chronic kidney disease promotes muscle inflammation through an upregulation of TLR4. TLR4 activation in muscle cells triggers an innate immune response mediated by NF-kB activation, downregulation of p-Akt and upregulation of the TNF-α-dependent inflammatory pathway [73].

The stress signaling is not exclusively generated by skeletal muscle cells. In mice, sciatic nerve denervation increases the levels of several TLRs and their downstream signaling adaptor myeloid differentiation primary response 88 (MyD88), leading to muscle atrophy. The TLR-MyD88 axis mediates skeletal muscle atrophy, potentially through the activation of canonical NF-κB signaling, AMPK and UPR pathways [74].

Overall, in the elderly, overactivation of PRRs and their signaling cascades leads to a chronic activation of the immune system, contributing to sarcopenia. Investigating the complexity of the different sensors and how they become activated will allow a better understanding of the downstream pathways that are triggered and their pathophysiological implications.

## 5. Cytokines/Myokines (*Friends or Foes*)

Cytokines are small soluble proteins which can act on the same cells that secrete them (autocrine action), on neighboring cells (paracrine action) or on distant cells (endocrine action). Cytokines are secreted by different cell types, and the ones produced by skeletal muscle are called myokines. The primary function of cytokines is to regulate inflammation, but they can also display different cell-type-specific functions and are therefore considered multifaceted molecules. In particular, myokines regulate with tissue regeneration, cell proliferation and differentiation, and their secretion can be exercise-associated.

Although classically categorized as pro-inflammatory or anti-inflammatory, cytokines present pleiotropic and dose-dependent effects with different outcomes if the secretion is transient or prolonged over time. In general, prolonged and elevated expression of cytokines and myokines in the context of advanced age exerts negative effects in sarcopenia [21]. While during muscle regeneration and repair, beneficial effects have been attributed to some of these molecules, for the elderly, elevated levels are mainly associated with sarcopenic profiles [75].

Dual effects have been characterized for TNF-α, interleukin-6 (IL-6) and fibroblast growth factor-21 (FGF21). Indeed, during early stages of muscle injury, short-term increases in TNF-α, considered a key mediator of the inflammatory responses and apoptosis, promote muscle repair [76,77]. However, continuous elevated levels of TNF-α lead to muscle damage [78], and increased levels of TNF-α are associated with the development of sarcopenia [79,80,81]. Interleukin 6, a proinflammatory cytokine that can also be secreted by myofibers and MuSCs, exerts a proliferative effect on the muscle stem cells [82]. However, chronic low levels of TNF-α in the elderly are associated with muscle atrophy [83]. Another myokine, Fibroblast Growth Factor 21 (FGF21), displays effects that are dose and age dependent, ranging from insulin-resistance protection to loss of muscle strength and unhealthy aging [84].

Two cytokines directly related to signs of infection or injury and loss of muscle mass are, respectively, C-reactive protein (CRP) [85] and growth differentiation factor-15 (GDF-15) [86]. CRP expression has been consistently related to sarcopenia [87], whereas the role played by GDF-15 still has to be clarified [88,89]. High levels of circulating pro-inflammatory cytokines (CRP, IL-6, TNF-α, etc.) have been found to correlate with low lower handgrip, decreased appendicular muscle mass and reduced knee-extension strength [87,90,91], indicating that these cytokines could be potential biomarkers of sarcopenia.

Pro-inflammatory cytokines interleukin-1 (IL-1) and IL-18 are secreted after inflammasome-dependent caspase-1 activation. Inflammasomes, in turn, are cytosolic multiprotein complexes containing PRRs activated by detection of stress molecules [92]. The inflammasomes activate Caspase-1, required for the cleavage of the inactive pro-IL-1β and pro-IL-18 molecules into their active counterparts. Active IL-1β has been associated with increased likelihood of all-cause mortality [93], and both IL-1 and IL-18 participate in the inflammatory aging process [94] and in sarcopenia [80,95].

Interestingly, interleukin-15 (IL-15) can positively regulate hypertrophy in myotubes and can even mitigate the inflammatory detrimental effects elicited by TNF-α [96]. Together with the finding that low levels of plasma IL-15 associate with sarcopenia in old people [97], these results highlight IL-15 as an interesting therapeutic target for sarcopenia.

Interleukin-10 (IL-10), although traditionally considered an anti-inflammatory cytokine [98], has pleiotropic functions that can be organ-, tissue- and disease-state-specific and that range from beneficial to maladaptive [99]. IL-10 knockout mice (IL-10^−/−^) have been used as a mouse model of sarcopenia, due to their reduced skeletal muscle mitochondrial function [100]. Consistently, overexpression of IL-10 ameliorates the aging-mediated skeletal muscle inflammation in insulin-resistant mice [101]. However, the implications of IL-10 regulation in the human elderly are yet to be defined.

IL-10 and IL-6 concentrations have been seen to be increased in patients with sarcopenia, as well as the IL-6/IL-10 ratio, suggesting that the increase in IL-10 might be a compensatory mechanism elicited to suppress the effects of increased IL-6 expression. These results indicate that the anti-inflammatory potential of IL-10 in elderly individuals with sarcopenia is not sufficient to overcome the basal pro-inflammatory status [102]. Nonetheless, elderly patients with Chronic Obstructive Pulmonary Disease diagnosed with sarcopenia presented high levels of IL-6 but not IL-10 [103].

Among the myokines secreted by skeletal muscle, Myostatin, Decorin and Irisin are the most studied, and they present opposite effects. Myostatin, a member of the TGF beta family, inhibits muscle differentiation and growth [104] by inducing cell-cycle arrest in satellite cells and myoblast [105] and inhibiting myoblast differentiation via downregulation of MyoD/Myogenin expression [106]. In opposition, Decorin, a myokine released in response to muscle contraction, directly binds Myostatin and regulates muscle hypertrophy by increasing expression of the myogenic factors MyoD1 and follistatin and reducing the atrophic markers atrogin1 and MuRF1 [107]. Physical activity is related to an increase in Decorin and decrease in Myostatin [108]. Nevertheless, the relationships of Myostatin and Decorin with sarcopenia are not clear, as some studies found association between these myokines and sarcopenia [109,110], whereas in others the association is unclear or is present in males but not in females [111].

Irisin has been identified as a myokine accelerating the browning of adipose tissue, improving obesity and glucose homeostasis [112]. It is involved in the downregulation of the insulin resistance pathway in a PGC-1α-dependent manner and leads to a protective effect against obesity and diabetes. Lower levels of Irisin are associated with muscle weakness and atrophy and with sarcopenia in patients with liver cirrhosis [113,114]. It has also been suggested that a crosstalk exists between Myostatin and Irisin, where downregulated levels of Myostatin promote browning of adipose tissue by increasing the levels of Irisin [115].

In general, the main mechanisms by which cytokines contribute to muscle dysfunction and sarcopenia are by interacting with the immune system [116] and by promoting a pro-inflammatory environment or by disrupting the oxidative stress balance [117]. Some discrepancies can be found among studies evaluating cytokines and myokines in the elderly. While some studies reported a significant correlation between the expression of these molecules and sarcopenic measurements, other studies failed to find a correlation [118]. This may be due to the different parameters studied to assess the severity of the sarcopenic phenotype (e.g., grip strength, knee extension, muscle mass, etc.), or to the difficulty related to sensitivity of the detection systems, especially for those cytokines expressed in a low concentration or with low stability. Although a beneficial function of cytokines after exercise has been reported, chronic expression leads to unwanted effects in the elderly and sarcopenic patients. Standardized parameters to measure sarcopenic levels and improving cytokine quantification techniques, together with a better understanding of the cytokine secretion dynamics, are essential to deeply understand the relationship of these molecules with the pathophysiological mechanism of sarcopenia.

## 6. Immunosenescence (*I Guess I Must Be Getting Old*)

The process of aging affects all organs and tissues, including the innate and adaptive immune systems. We refer to the changes of the immune system occurring with advanced age as immunosenescence [119]. These age-associated alterations affect hematopoietic stem cell (HSC) function, exhibiting reduced homing and differentiation capacity, alterations in natural killer (NK)-cell number and function, impaired B-cell development and thymic involution, leading to a decline of naïve T cells and to a decrease in T cell receptor (TCR) repertoire and signaling. [120]. The disruption of the crosstalk between the immune system and muscle cells likely contributes to the decrease in muscle regeneration with age.

Muscle regeneration is a highly synchronized process involving satellite cell activation, proliferation, differentiation and fusion [121]. Tight coordination between inflammation and regeneration is crucial for the repair process following muscle damage or renewal. Thus, the extracellular niche environment becomes essential to complete the skeletal muscle dynamics. The first action comes from pro-inflammatory macrophages stimulating the proliferation of MuSCs. For appropriate differentiation of MuSCs into myofibers, a shift from a pro-inflammatory to an anti-inflammatory phenotype of immune cells is required [122,123]. Disruption of this sequence leads to impairments of the muscle reparation mechanisms and MuSCs’ self-renewal.

The initial cellular infiltration in the lesion site is composed of mast cells and neutrophils. In aged rats, it has been demonstrated that the number and volume of mast cells, a source of tumor necrosis factor-α (TNF-α), are both increased compared with young rats after an ischemia–reperfusion injury model [124]. Importantly, neutrophils involved in phagocytosis and chemotaxis play an essential role in the process of regeneration, since neutrophil-depleted mice display incomplete muscle recovery and unresolved necrotic fibers [125]. In humans, it was reported that a decrease in neutrophil number correlated with aging [126].

In a subsequent phase, circulating monocytes migrate from blood to the site of the lesion and differentiate intro M1 macrophages. These macrophages are characterized by a clear pro-inflammatory phenotype, with expression of CD68 and iNOS (inducible nitric oxide synthase), and secrete cytokines/chemokines. M1 macrophages are in charge of lysis and phagocytosis of injured cells and cellular debris [48]. Additionally, M1 macrophages have a dual role, attracting satellite cells to the injured site and stimulating their proliferation [127]. Conversion from a pro-inflammatory M1 to an anti-inflammatory M2 phenotype is essential for differentiation of muscle cells. This conversion is mediated by an increase in anti-inflammatory cytokines, such as TGF-β and IL-10, and a decrease in the pro-inflammatory cytokines, such as IFN-γ, leading to the expression of different markers such as CD206 and CD163 in these muscle cells. Therefore, macrophage conversion from a pro-inflammatory M1 to an anti-inflammatory M2 phenotype plays a pivotal role in muscle regeneration. Another particularly important step is the complete inactivation of macrophages and cytokine secretion after the end of muscle repair [128]. Therefore, the unresolved switch of macrophages after repair can lead to a muscle chronic inflammatory phenotype.

During aging, monocytes, macrophages and cytokine secretions are altered, and the crosstalk with skeletal muscle cells can be affected. These relationships have been shown in the muscles of elderly men containing less total number of macrophages, and in particular, less pro-inflammatory macrophages than those in young men, whereas the number of anti-inflammatory macrophages remained unchanged compared to young muscle [129]. After resistance exercise, only in muscle from young subjects was there an increase the number of both anti- and pro-inflammatory macrophages [129], and the expression of pro-inflammatory cytokines MCP-1, IL-6 and IL-8 was higher in old compared to young adults [130]. Therefore, aging results in a defective regulation of muscle macrophage function.

In addition to macrophages, regulatory T cells (Treg) promote the M1-to-M2 switch and the activation of satellite cells via the secretion of specific cytokines, such as Interleukin-33 (IL-33) and by releasing growth factors, such as amphiregulin [131]. In old mice, there is a decrease in the accumulation of Treg cells after muscle injury owing to defects in multiple aspects of their population dynamics: recruitment, proliferation and retention [132].

To demonstrate cell-specific accelerated aging of the immune system drives senescence and loss of tissue homeostasis in non-lymphoid organs, a mouse model of defective DNA repair restricted to hematopoietic stem cells (HSCs) was used; specifically, the Ercc1 knockout line [133]. These mice showed accumulation of DNA damage and early senescence in immune cells with increased p16 and p21 expression [133]. Ercc1-deficient mice display accelerated senescence and widespread tissue damage upon aging, which is not restricted to HSCs lineage. These results stress how aging limited only to the immune system causes systemic aging by inducing senescence and damage in non-lymphoid organs.

We can conclude that immune dysfunction associate with age leads to changes in the immune system, affecting systemically and is related with several pathologies including sarcopenia. The tight coordination of muscle regeneration with the immune response becomes altered in aging impairing normal muscle homeostasis and leading to atrophy.

## 7. Gut and Metabolic Disorders: Metabolic Syndrome (*Toxic Sugar*)

The drastic changes in gut microbial composition occurring with aging are known as microbiome dysbiosis and have been associated with non-gut-associated diseases [134,135]. During aging, the decrease in the beneficial commensal gut microorganisms is concomitant to an expansion of potential pathogenic and pro-inflammatory microorganisms [136]. The disturbance of the crosstalk between gut microbiota and the host due to age-related gut dysbiosis can affect the host health and lifespan [137]. Additionally, it has been shown that older mice have thinning intestinal barriers facilitating bacterial penetration and release of DAMPs, increasing the production of inflammatory mediators, including TNF-α [138].

The interaction between skeletal muscle and gut microbiota dubbed the “gut–muscle axis” could be a reason behind sarcopenia. For instance, patients with inflammatory bowel disease (IBD), a chronic gut inflammatory disease of the intestinal tract, present significant changes in the gut microbiota associated with an increase in intestine permeability. Studies reported that, compared with healthy people, IBD patients are more likely to have sarcopenia [139]. In induced-colitis murine models, muscle atrophy has been related to an increased breakdown of proteins mediated by the upregulation of the proteasome and enhanced expression of skeletal-muscle-atrophy-related genes Atrogin-1 and Murf-1 together with a downregulation of IGF1-R and phospho-mTOR [140]. In patients with Crohn’s disease, reduced muscle mass and atrophy have been associated with impaired activation of protein synthesis pathways, indicated by a lower ratio of phosphorylated Akt, potentially mediated by elevated levels of proinflammatory cytokines and reduced IGF-1 levels [141]. Specifically, assessing gut microbiota composition in physical frailty and sarcopenia is key to understanding the pathophysiology of the gut–muscle axis.

Metabolic syndrome (Met-S) includes a concomitant group of conditions that, together, raise the risk of coronary heart disease, type 2 diabetes, stroke and other health issues. These conditions include abdominal obesity, insulin resistance, abnormal cholesterol or triglyceride levels, dyslipidemia, inflammation or hypertension. Associations between sarcopenia and metabolic syndrome have been reported [142] and concurrent pathologies present an increased risk of adverse health events [143]. Chronic inflammation, a sign of Met-S, could be a possible link with sarcopenia. One of the main causes of Met-S is increased insulin resistance (IR) [144]. Skeletal muscle is the main peripheral tissue implicated with insulin resistance in type 2 diabetes, mainly due to decreased stimulation of glycogen synthesis in muscle by insulin [145]. Hyperinsulinemia caused by IR also increases the amount of myostatin, a biomarker of muscle wasting [110], and decreases protein synthesis by inhibiting the growth hormone insulin-like growth factor 1 (IGF1) pathway [146]. Low levels of persistent inflammation in diabetes patients [147,148] have been correlated with the activation of the p38 MAPK pathway [149] and have been shown to be responsible for diabetes-associated muscle atrophy [150]. This, in addition to an impaired bioenergetic capacity measured as NADH:O(2) oxidoreductase activity and citrate synthase activity in skeletal muscle mitochondria of diabetes patients, may be responsible for the disruption of muscle homeostasis in this condition [151]. In both humans and rodents, IR have been related to an increase in the rate of mitochondrial H_2_O_2_ emission and redox imbalance in skeletal muscle, linking mitochondrial bioenergetics to the etiology of insulin resistance [152]. Sirtuin 2 (Sirt2), a NAD-dependent protein deacetylase that participates in deacetylation of histones and other proteins, also contributes to insulin resistance and metabolic syndrome by regulating NLR family pyrin domain containing 3 (NLRP3)-mediated inflammasome activation [153].

Taken together, gut microbial dysbiosis and metabolic disorders highlight the importance of the gut–muscle axis and how gut inflammation strongly correlates with sarcopenia.

## 8. Adipose Tissue Implications (*Toxic Energy*)

The role of the adipose tissue in inflammation and aging is closely linked with metabolic disruptions. Excessive or dysfunctional adipose tissue appears to accelerate the onset of multiple age-related diseases [154]. Upon aging, adipose tissue is redistributed outside fat depots, accumulating viscerally. In particular, in skeletal muscle tissue, it can be found accumulated as ectopic fat depots or as intramuscular lipid droplets.

Adipocytes infiltrated inter- or intramuscularly are prone to generating a pro-inflammatory environment. Adipose tissue expression of pro-inflammatory cytokines such as TNF and IL-6 promotes obesity-related insulin resistance in humans [155] and the secretion of these molecules is often increased with aging [156,157]. Additionally, local adiposity promotes the attraction of immune cells by the recruitment of macrophages. Adipose tissue macrophages generate cytokines that promote insulin resistance and muscle atrophy and contribute to the feedback loop by promoting fat accumulation in non-adipose tissue [143]. Aging can contribute to this process by increasing the abundance of macrophages in subcutaneous fat, as observed in old mice [158], and by altering the balance of visceral adipose tissue macrophages toward the pro-inflammatory M1 phenotype [159].

Adipocytes also act as endocrine cells, secreting adipokines and contributing to inflammation. Age-related changes in the levels of myokines, such as myostatin and irisin, and adipokines, such as leptin and adiponectin, can regulate the sarcopenic phenotype [160]. The same holds true for changes in expression of pro-inflammatory adipokines, such as leptin, and anti-inflammatory adipokines such as adiponectin. Age-related changes in fat tissue include an increase in senescent or senescent-like cells, contributing to age-related fat tissue inflammation and dysfunction [161,162].

Furthermore, excessive lipids in the form of free fatty acids (FAs) induce mitochondrial dysfunction, disturb β-oxidation of FAs and enhance reactive oxygen species (ROS) production, resulting in autophagy/apoptosis of muscle cells and leading to lipotoxicity, impaired myokine release and insulin resistance [163].

Fibro–adipogenic progenitors (FAPs) are an interstitial mesenchymal cell population of skeletal muscle with the ability to differentiate into fibroblasts and adipocytes, but not into myoblasts, and they also have the capacity to activate satellite cells upon injury [164]. In obesity and in type 2 diabetes, FAPs can cause chronic inflammation, fibrosis, intramuscular fat accumulation in skeletal muscle and muscle fiber degeneration by secreting pro-inflammatory cytokines as IL-6 [165,166,167]. Additionally, FAPs can be activated by myokines [168]. Therefore, the cross-talk between the immune system and FAPs is essential for muscle repair [169].

Body composition changes during the aging process due to hormonal, inflammatory and myocellular mechanisms which increase the prevalence of obesity and sarcopenic obesity in an aging population [170]. This natural tendency, together with increased lack of regular exercise and high caloric intake, are core factors underlying sarcopenic obesity. In a multi-continent study, the percentage of body fat has been identified as a key factor for the prevention of sarcopenia syndrome [171]. Moreover, the concept of metaflammation can be conceived as a specific type of chronic inflammation caused by nutrient excess [26]. This notion emerged from the idea that over-nutrition, which is present in metabolic diseases such as obesity and type 2 diabetes, can drive into chronic inflammation [172].

Leptin-deficient animals, referred as ob/ob, are characterize by hyperphagia, reduced energy expenditure and extreme obesity [173]. Studies using this mouse model found that leptin-deficient obese mice adipocytes show a hypertrophic phenotype with ultrastructural changes accompanied by oxidative stress via NLRP3-dependent caspase-1 activation [174]. Additionally, increased oxidative stress status, blockade of autophagy, activated unfolded protein response, exacerbated inflammatory response and impaired insulin signaling are common features in these mice [175]. Melatonin could significantly reduce endoplasmic reticulum stress and adipogenesis through autophagy modulation in the liver of leptin-deficient obese animals [175,176] and ameliorate oxidative stress, improving mitochondrial respiration and decreasing protein aggregates level in the brain of these mice [177,178].

All these data indicate the influence of increased adipose tissue on inflammation via pro-inflammatory cytokines and adipokines, the consequent attraction of macrophages, along with mitochondrial dysfunction and oxidative stress, enhancing the aging and sarcopenic phenotype.

## 9. Autophagy and Oxidative Stress Implications (*Destroy Everything You Touch*)

Protein turnover is especially essential for high-dynamic tissues such as skeletal muscle. Cell contraction requires high oxygen consumption and continuous ATP supply to the skeletal muscle contractile apparatus involves mitochondria biogenesis. The ubiquitin–proteasome and the autophagy–lysosome systems are the main pathways involved in protein turnover in the muscle. The proteasome system provides rapid elimination of single proteins or small aggregates by two ubiquitin ligases, namely atrogin-1 and MuRF1. On the other hand, the autophagy system can engulf a portion of cytoplasm to degrade entire organelles and recover the amino acids derived from protein degradation [179]. During aging, the protein turnover is slowed down and aggregates of dysfunctional proteins are accumulated at the cytosol impairing the normal functions of the cell [180]. Muscle-specific deletion of a crucial autophagy gene, Autophagy-related 7 (Atg7), causes age-dependent muscle atrophy in mice, coupled with accumulation of abnormal mitochondria, sarcoplasmic reticulum distension and disorganization of sarcomere [181]. The Atg7 conditional knockout line also serves as a model of sarcopenia, validating the contribution of basal autophagy in maintaining myofiber integrity [181].

Autophagy has important effects on the induction and modulation of the inflammatory reaction. If autophagy is defective, it leads to an accumulation of depolarized mitochondria that release inflammasome activators such as reactive oxygen species (ROS) or mtDNA. Mitochondrial function in skeletal muscle is known to decline with age, impairing the ability to oxidize metabolic fuels in mitochondria, reducing ATP levels and altering the endogenous levels of ROS, thus leading to cumulative damage during aging [182]. Autophagy disruption can trigger inflammation through two different pathways. First, increased ROS production promotes inflammation mainly via NF-κB (nuclear factor κ-light-chain-enhancer of activated B cells) by way of nuclear translocation and signaling activation [183,184]. Additionally, muscle cells need an efficient system to eliminate damaged proteins and dysfunctional mitochondria, with autophagy being the main pathway responsible for mitochondrial turnover [185].

Autophagy has other important effects on the induction and modulation of the inflammatory reaction by altering the metabolic status during immune-cell differentiation through AMPK and mTOR signaling cascades. mTOR activation leads to reduced autophagic flux, increased glycolytic activity and transitioning of macrophages to a pro-inflammatory and proliferative state, whereas AMPK activates autophagy by an OXPHOS-dependent mechanism promoting the transitioning into a quiescent and anti-inflammatory state [186]. During aging, defective autophagy influences macrophage polarization and cytokine release, increasing the levels of pro-inflammatory mediators and activating a senescence-associated secretory phenotype, which promotes M1 macrophage polarization [187]. The essential role of autophagy in preventing immunosenescence can be observed in Atg7-deficient mice, where macrophage populations are increased, their function reduced and the inflammatory cytokine response enhanced, resembling aged macrophages [188]. In particular, in disused skeletal muscle, muscle regrowth is impaired by loss of classical M1-to-M2 macrophage transition patterns during recovery [49]. Furthermore, in the soleus muscle, reduced numbers of macrophages are found in early stages of recovery compared to young animals [49]. All these data highlight the importance of the fine-tuning between autophagy and inflammation.

The connections between autophagy and immunity are even more intermingled, since autophagy is considered to have a crucial role in the defense against pathogens [189]. There is a feedback loop in the interplay between autophagy and inflammation: autophagy can be induced by immune signals and inflammation can be triggered by autophagy. PRRs have been reported to induce both autophagy and inflammation concomitantly. Cytokines including TNF-α, IL-1 or IL-6 have been shown to induce autophagy, while others such as IL-10 and IL-13 have inhibitory effects. In turn, autophagy itself regulates the production and secretion of cytokines, including IL-1, IL-18, TNF-α and Type I IFN [190]. In a similar manner, autophagy proteins can both activate or inactivate the innate immune signaling [191]. Autophagy can reduce inflammation by eliminating damaged mitochondria, reducing concentrations of ROS, increasing clearance of DAMPs and therefore preventing the inflammasome activation. Paradoxically, autophagy can also induce activation of the innate immune response by promoting TLR signaling in the presence of vesicle-bound nucleic acid. Autophagy also inhibits IL-1β secretion by repressing inflammasome activation. Basal mitophagy/autophagy negatively regulates inflammasome activity mediated by NLRP3 [192] and NLRP4 [193] but, conversely, ROS-induced autophagy positively regulates the production of IL-1β [194].

In aging muscle, there is a reduced mitochondrial volume and reduced oxidative capacity [195] leading to a state of oxidative stress capable of triggering an inflammatory response. Reduced autophagy with aging may be the cause underlying the age-associated decline in muscle stem cells pool. Autophagy is a key factor in the conversion from quiescent to senescent muscle stem cells. A decline of autophagy in satellite cells results in toxic cellular waste accumulation, which triggers entry into senescence and is associated with increased ROS levels [196]. Likewise, sarcopenic patients with loss of independent motor function exhibit a decrease in protein synthesis associated with increased response to misfolded or unfolded proteins, autophagy blockade, as well as increased oxidative stress and increased apoptosis mediated by Caspases 3 and 7 [185]. In the elderly, mitochondrial dysfunction together with insufficient autophagy may promote a pro-inflammatory environment, worsening sarcopenia outcomes [197,198].

## 10. Interventions

### 10.1. Senolytics (Eliminate the Wrong)

Sarcopenia has been reported to be more influenced by the development of a senescence-associated secretory phenotype (SASP) rather than a decrease in the satellite cell population number [199]. In order to restore muscle homeostasis, clearing of senescent cells by senotherapeutic compounds that selectively kill senescent cells (senolytics) or suppress the SASP (senomorphics) could be a strategy to improve or prevent deterioration of skeletal muscle during aging. Senescent transplanted cells have the potential to cause physical dysfunction and spread a senescent environment, even if they are transplanted to young mice [200]. Pharmacological and genetic approaches to eliminate senescent cells improved muscle performance and survival. Elimination of senescent cells through oral administration of a senolytic cocktail in both naturally aged mice and senescent cell-transplanted young mice was able to improve physical dysfunction and reduce mortality [200]. Nuclear exclusion of p53 in senescent cells by administration of a FOXO4-p53-interfering peptide improved the physical performances of aged mice [201]. Eliminating senescent cells decreases the risk of chemotherapy-induced fatigue, improving physical activity and strength in a mouse model of cellular senescence (p16-3MR mice) and in human patients [202]. Senescent cells are resistant to apoptosis. Administration of the senolytic pharmacological agent ABT263, which selectively kills senescent cells by inducing apoptosis, efficiently depleted senescent muscle stem cells [203]. Additionally, genetic inducible elimination of p16^Ink4a^-positive senescent cells in a progeroid mouse model delayed age-related pathologies in skeletal muscle and adipose tissue [204].

There is a great potential in targeting senescent cells to ameliorate skeletal muscle inflammaging and improve health and lifespan. To further understand the role of senescence in skeletal muscle, cell-type-specific genetic depletion of senescence-related genes in satellite cells or in macrophages could disclose the particular implications of senescence in these cell types and its involvement in sarcopenia. Along the same lines, tissue-specific delivery of senotherapeutic drugs may help to avoid offside effects, as senescence has been associated with limit tumorigenesis and tissue damage in specific tissue environments [205].

### 10.2. Melatonin (The Many Roads of Melatonin: Anti-Inflammatory Effect)

Melatonin (N-acetyl-5-methoxytryptamine) is an evolutionary-conserved molecule considered a regulator of circadian rhythms among other pleiotropic functions [206]. Additionally, melatonin has multiple functions as an antioxidant and free radical scavenger, and as an immune-modulator and endocrine-modulator molecule [207,208]. Together with the fact that melatonin levels progressively decrease with age, this suggests involvement of melatonin in age-associated diseases and specifically in inflammaging [209,210]. In fact, low levels of melatonin are measured in patients with Parkinson’s disease, Alzheimer’s disease, type 2 diabetes, osteoporosis, rheumatoid arthritis, ischemic injury, and neuropsychiatric disorders [211,212,213]. Furthermore, melatonin levels are decreased in diseases related to insulin resistance [214]. Melatonin supplementation can improve metabolic syndromes by attenuation of inflammatory responses [215] and regulating autophagy [216]. The regulatory role of melatonin in the autophagic mechanism appears to be the protection of the membrane potential and mitochondrial DNA damage. Melatonin triggers deacetylation of silent information regulator 1 (Sirt1) and restores mitochondrial metabolism, whereas at the same time it protects against autophagic cell death by mediating the dissociation of the Beclin1-BCL2 complex [217]. Beneficial effects against mitochondrial dysfunction have been reported, too, as melatonin can influence mitophagy, the autophagy mechanism to remove damaged mitochondria [218].

Melatonin and leptin are involved in common processes, such as adipose tissue regulation and chronic inflammation [219]. Melatonin increases leptin concentration after oral administration [220] and reduces adipogenesis, endoplasmic reticulum stress and autophagy in leptin-deficient mice [175,176]. Melatonin also displays immunomodulatory roles by increasing cellular and exosomal α-ketoglutarate level in adipose tissue and alleviating metabolic inflammation [221]. Anti-inflammatory actions of melatonin in aging also include downregulation of proinflammatory cytokines observed in aged mice after femoral artery ligation, ovariectomized female rats or aging rats [222].

Melatonin has been proposed to prevent or resolve sarcopenia-associated diseases as it counteracts mitochondrial impairments, reduces oxidative stress and autophagic alterations in muscle cells, increases the number of satellite cells and limits sarcopenic changes in skeletal muscle [223]. Muscle aging hallmarks, such as mitochondria ultrastructure, lactate production, muscle fiber number, apoptotic nuclei number or age-associated myofibrils damage, are all ameliorated after oral administration of melatonin [224].

Aged mice treated with melatonin show increased muscle mass and fiber numbers, as well as an improvement in muscle architecture, together with a decrease in fibrosis and apoptosis. Melatonin also prevents age-associated mitochondrial damage due to oxidative stress and can reverse the deleterious effects of age-associated overactivation of the NLRP3 inflammasome [225]. This effect of melatonin is potentially mediated by two mechanisms. The first is due to its antioxidant and ROS-scavenging effects [226] and, additionally, its anti-inflammatory effect which inhibits the expression of iNOS (inducible nitric oxide synthase) [227], another NLRP3 inflammasome activator.

The best therapeutic melatonin dosage in human diseases is still under debate [228]. Some studies pointed out that the benefits of melatonin are strictly time dependent, dose dependent and exercise dependent [229]. Although more studies are needed to elucidate this point, melatonin potentially could help ameliorate inflammaging by (1) autophagy and mitophagy regulation, (2) oxidative stress reduction, (3) anti-inflammatory properties, (4) regulation of leptin expression and (5) regulation of insulin resistance and of adipose tissue accumulation.

## 11. Conclusions (*Aging Is the Only Way of Living*)

Inflammation is an evolutionarily conserved mechanism protecting organisms against infections and injuries. The immune system increased in complexity with evolution to protect individuals and species against a large range of adverse conditions, but dysfunctions of this system represent a double-edged sword and are probably responsible for many contemporary diseases [230]. Appropriate inflammation helps muscle repair, but excessive or low and continued inflammation will further promote injury and cell death. Fine-tuning of the immune response becomes essential due to the complexity of the biological needs. Undercovering the mechanisms regulating and deregulating immunity, especially during aging, becomes a demand in an increasingly aging world.

Long-lived people, in particular centenarians, can be a model of successful aging with a low rate of age-related diseases and frailty. One of the major reasons for this resilience has to be the capacity to counterbalance the age-associate inflammation with efficient anti-inflammatory responses, known as “anti-inflammaging”. This predisposition to an anti-inflammatory environment could be the key to longevity. The capacity to outweigh the pro-inflammatory cytokines by balancing their effects with anti-inflammatory stimuli could be the way to adapt to aging life and its correspondent age-associated diseases [231]. Centenarians alleviate inflammaging by decreasing the ratio and secretory profile of T Helper 17 and Regulatory T Cells (Th17/Treg) into anti-inflammatory secretory phenotypes [232].

**Figure 1:**
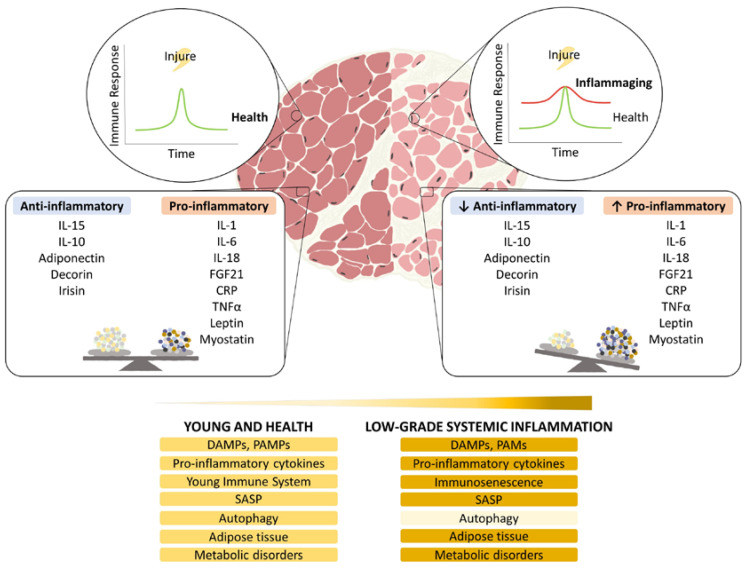
Overview of the main factors affecting aging associated inflammation in sarcopenia.

Activation of subclinical, chronic low-grade inflammation occurring with aging should be taken into consideration, as growing studies are relating this phenomenon to multiple age-associated diseases, including sarcopenia (Figure 1). Autophagy-based control of inflammation through maintenance of mitochondrial homeostasis and suppression of ROS release may be a promising strategy for preventing the negative effects associated with inflammasome activation and keeping a healthy anti- and pro-inflammatory balance.

## 12. Future Directions (*Save Myself*)

Understanding the precise interplay between chronic low-grade inflammation and the risk of age-related pathologies should be implemented as a strategy to promote healthy aging in the general population. Development of clinical applications that promote healthy lifespan would include lifestyle interventions, including an anti-inflammatory dietary approach (preventing metaflammation), regular exercise and maintaining healthy behaviors, understood as non-pharmacologic approaches. Pharmacological interventions aimed at controlling inflammation (such as autophagy activation, ablation of senescent cells, SASP interference, etc.) should be initiated in the experimental animal phase and, subsequently, in clinical trials.

Future research should focus on metabolomics, lipidomics and gut microbial composition to provide biomarkers at the interface between metabolism and inflammation. Secretome analysis would unravel the complete set of proteins secreted by different cell types present in the skeletal muscle and neighboring tissues such as adipose tissue. After characterizing them, the specific targeting of cytokines, adipokines and myokines could represent a strategy to restore the aging imbalance.

To understand the pathophysiological mechanism of sarcopenia and the mechanisms triggering this state, tissue-specific determination of inflammatory pathways due to the cell/tissue specificity of the immune system should be addressed. The inflammaging machinery displays a bow tie architecture [233] with a fan-in (inputs), a knob or core (an evolutionary limited repertoire of blocks), a fan-out (downstream effects) and feedback loops. The fan-in is represented by a broad spectrum of exogenous and endogenous danger stimuli (DAMPs or PAMPs), which activate a limited number of evolutionarily conserved innate immunity sensors (core), whose activation produces a large number of inflammatory compounds (fan-out) [26]. While the core has a conserved mechanism among different species which is shared by different cell types, the inputs and outputs can be cell-type specific. Therefore, the need arises to study these mechanisms and their particularities in the context of skeletal muscle aging and sarcopenia.

Studies in models with accelerated muscle aging could complement the human studies in order to find the best approach to prevent inflammmaging. Interventions could start by quenching PRRs using agonists, reducing DAMPs by increased autophagy to control excessive ROS production or administrating antioxidants. Senotherapeutic drugs to eliminate senescence cells or immunomodulatory drugs to keep the cytokine balance could also be other strategies to be used.

## Data Availability

Not applicable.
